# Magnitude of self-harm and associated factors among postnatal mothers attending immunization clinics at public health facilities in Boneya Boshe Woreda, Western Ethiopia, 2023: institution-based cross-sectional study design

**DOI:** 10.3389/fpubh.2024.1384688

**Published:** 2024-05-16

**Authors:** Lema Fikadu Wedajo, Mohammedamin Hajure, Zakir Abdu, Gebremeskel Mulatu Tesfaye, Yadeta Alemayehu Workneh, Wubishet Gezimu, Mustefa Adem Hussen, Aman Dule Gemeda, Sheleme Mengistu Teferi, Solomon Seyife Alemu

**Affiliations:** ^1^Wallaga University Institute of Health Sciences, Nekemte, Ethiopia; ^2^Madda Walabu University College of Medicine and Health Sciences, Robe, Ethiopia; ^3^Mattu University College of Health Sciences, Mattu, Ethiopia

**Keywords:** self-harm, immunization clinics, Boneya Boshe, infant immunization, postnatal mothers

## Abstract

**Background:**

Self-harm is a preventable, but a leading, cause of maternal morbidity and mortality all over the world, with a significant impact on healthcare systems.

**Objective:**

To assess the magnitude of self-harm and associated factors among postnatal mothers attending immunization clinics.

**Methods:**

An institution-based cross-sectional study was employed among postnatal mothers attending infant immunization clinics at public health facilities in Boneya Boshe Woreda, Western Ethiopia, 1 October to 30 October 2023. A pretested, face-to-face interviewer-administered structured questionnaire prepared by Kobo Toolbox was used to collect the data. Both bivariable and multivariable logistic regression analyses were done. The level of significance was declared at *p*-value <0.05 with a 95% CI.

**Results:**

Among the 423 mothers enrolled in the study, 415 of them finally participated, at a response rate of 98.10%. The magnitude of self-harm was 12.53% (95% CI: 9.33, 15.73). Involvement of husband in maternity and child healthcare (AOR = 1.90; 95% CI: 1.12, 2.10), depression (AOR = 2.79; 95% CI: 2.14, 6.94), loneliness (AOR = 2.49; 95% CI: 1.15, 5.40), postpartum intimate partner violence (AOR = 2.15; 95% CI: 1.01, 4.54), average monthly income (AOR = 3.70; 95% CI: 2.17, 10.50), and postnatal care (AOR = 2.72; 95% CI: 1.28, 5.80) were significantly associated factors.

**Conclusion and recommendations:**

The study sought a magnitude of self-harm that was slightly higher than the previous study conducted in the northern part of Ethiopia. Therefore, healthcare providers should focus on identified factors during postnatal care to overcome them. Similarly, the concerned body should develop an effective strategy based on the identified factors to pay attention to postnatal mothers.

## Introduction

Self-harm is an intentional in which a person causes harm to their own selves as a coping mechanism when gripped by difficult or distressing thoughts and feelings. It most frequently takes the form of cutting, burning, or non-lethal overdoses. However, it can also be any behavior that causes injury to the victim, whether it is low or high (1, 2). The term self-harm encompasses a broad spectrum that includes a wide range of behaviors and intentions, including attempted hanging, impulsive self-poisoning, and superficial cutting in response to intolerable life events in the life process (3).

Self-harm is one of the most common reasons for hospital visits. It is a reflection of distress rather than a diagnosis in itself and is currently increasing among women of reproductive age, particularly during the perinatal period (4, 5). This problem is more common in women than men, which is one of the current global health challenges across the world (6).

During perinatal period, women are at the greatest risk of mood changes as a result of hormonal changes and life stress events related to pregnancy and childrearing processes (7–9). Common mental health problems like suicidal behaviors, postpartum psychological distress, anxiety, and postpartum stress disorders can lead to self-harm during the postnatal period if not identified and managed at an early stage (10–12).

Mental health problems are one of the leading global causes of maternal morbidity and mortality, posing the greatest challenge throughout the world (13). They also manifest as self-harm or thoughts of self-harm. The World Health Organization (WHO) estimates the magnitude of self-harm among the general population at 75.5% in both low-and middle-income countries (14). The studies conducted on postpartum mothers in both low-and high-income countries identified a varied magnitude of the problem that ranges from 4.6 to 27.4% (9, 15–17).

As identified by previous research, mental disorders, substance misuse, younger age, being unmarried, and obstetric and neonatal complications were factors leading to self-harm among postnatal mothers (18–20). In addition, lack of social support, lack of emotional support, and intimate partner violence were the other identified factors (5, 21).

Self-harm has significant consequences for infants, family members, and the healthcare system. In addition, it affects the Sustainable Development Goal agenda, which focuses on ending preventable maternal deaths by 2030 (22). Maternal self-harm leads to poor maternal–infant bonding processes that might affect infant health (15, 23). Similarly, the study identified that maternal self-harm thought causes self-harm thought in the offspring in the future and leads to early discontinuation of breastfeeding, which might increase childhood morbidity (24, 25). Maternal self-harm thoughts can also lead to childhood self-harm and suicidal ideations (26).

During the postnatal period, mothers may suffer from life-threatening health problems, including mental health problems. Even though this problem has a multi-dimensional impact, little attention has been given to it in both high-and low-income countries, which can lag behind the sustainable development goal three that focuses on the eradication of preventable causes of maternal deaths. Little attention has been given to maternal deaths all over the world, including in this specific study area, which is the focus of our research.

Therefore, this study aimed to assess the magnitude of self-harm and associated factors among postnatal mothers attending immunization clinics in public health facilities in Boneya Boshe Woreda, Western Ethiopia, in 2023. The findings of this study can be used as an input by healthcare providers to provide an evidence-based care plan to alleviate it. In addition, it may help the government to prevent maternal deaths related to self-harm and may be used as an input by scholars for further research.

## Methods and materials

### Study design, area, and period

An institution-based cross-sectional study design was conducted in Boneya Boshe Woreda from 1 October to 30 October 2023. This Woreda is found in the East Wallaga Zone of the Oromia region, which is located 81 km from the capital city of the zone, Nekemte town, and 311 km from the capital city of the country, Addis Ababa. Boneya Boshe Woreda is bordered by Nono Benja Woreda in the south, Gobbu Sayo and Sibu Sire Woreda in the north, Bako Tibe and Ilu Galan in the east, and Wahama Hagalo in the west.

In this Woreda, the total population is 73,227, with 14,925 households in 12 Kebeles. Out of this population, 34,803 are in the reproductive age group, of which 16,206 are women in the reproductive age group. This Woreda has an expected delivery rate of 2,541 per year. The 2022 Woreda Health Bureau report revealed that the total delivery in one year was 2,355. This Woreda has three health centers and 12 health posts, with a total of 68 health professionals to carry out healthcare activities.

### Eligibility criteria

All postpartum mothers attending immunization clinics in the first one year after childbirth during data collection period and who presented with their infants whose age was from two weeks after birth to one year were included in the study. However, those mothers who were critically ill during the data collection period and not permanent residents of this Woreda were excluded from the study.

### Sample size determination and sampling technique

The sample size was determined by using a single population proportion formula based on assumptions of 95% CI, 5% margin of error, and 50% of the proportion of self-harm since there has been no study done on this population in the study area, as follows:

$$n=\frac{\left(Z\frac{\propto }{2}\right)2pq}{d2}\text{.}$$

Then

$$n=\frac{\left(1.96\right)2\left(0.5x0.5\right)}{\left(0.05\right)2}=384.$$

**Figure 1:**
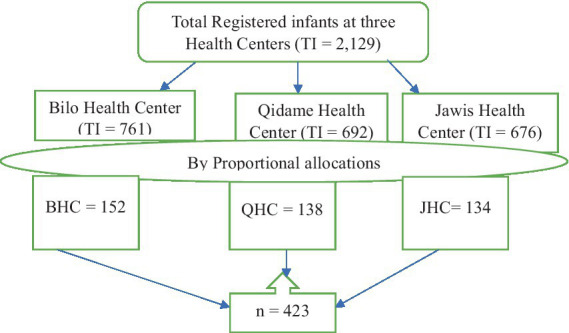
Diagrammatic presentation of sampling procedure to assess the magnitude of self-harm among postnatal mothers in Boneya Boshe Woreda, 2023. BHC, Bilo Health Center; QHC, Qidame Health Center; JHC, Jawis Health Center; TI, Total Infant.

Finally, by adding 10% of the non-response rate, the final sample size of 423 was arrived at for this study. A systematic random sampling technique was used to select the study participants based on the total number of infants attending immunization clinics. Initially, the total number of infants attending three immunization clinics in this Woreda was obtained from the infant immunization logbooks of each health center. Those health centers are Bilo Health Center, Qidame Health Center, and Jawis Health Center. The number of infants attending immunization clinics were 761 from Bilo Health Center, 692 from Qidame Health Center, and 676 from Jawis Health Center 676. Then it was proportionally allocated, and the allocated mothers were selected by a systematic random sampling method as follows (Figure 1).

## Variables of the study

### Dependent variable

The magnitude of self-harm was the dependent variable in this study.

### Independent variables

Socio-demographic variables include maternal age, husband’s age, maternal educational level, husband’s educational level, maternal occupation, husband occupation, average monthly income, and family size.

### Medical, reproductive history, and substance-related factors

The data collected for the study include the place of delivery, postnatal follow-up, history of adverse pregnancy outcome, maternal history of known diagnosed medical illnesses, history of diagnosed mental health problems during pregnancy, husband’s involvement in maternity and child healthcare, husband’s index of pregnancy during the current baby, husband’s satisfaction with the gender of the current baby, alcohol use by the father and mother of the infant, husband’s khat use, and husband’s smoking habit.

### Psychosocial-related factors

Intimate partner violence during the postnatal period, postpartum depression, maternal social support level, loneliness, and the decision-making power of the mother were explored.

### Operational definitions and measurements

Intentional self-harm is the condition in which an individual tries to hurt themselves purposefully. They could indulge in self-injurious behaviors like cutting, burning, hitting, hanging, overdosing, poisoning, banging head on objects, scratching the body, bloodletting (intentional act of an individual to let blood from them for self-hurt), strangulating the neck (the act of an individual to forcing or damaging one’s own neck or body for self-hurt), and electrocuting by the mother who intended to harm herself. If she has at least attempted one or more items developed provided in the Self-harm Screening Inventory (SHSI) tool to hurt herself during her first year after childbirth, she is considered to have done intentional self-harm, for which code “1” was assigned. For a mother who has not tried any of the items mentioned in the SHSI tool is considered to have not resorted to intentional self-harm, which was coded as “0” (27).

*Partner involvement in maternity and child healthcare*: This variable was assessed with nine questions. For each question, the responses were scored between 0 and 1. The total score was 9, with a minimum of 0 and a maximum of 9. Hence, husband’s involvement with a score above the median showed that the husband was involved in maternity an child healthcare (28).

*Postpartum depression*: The women who had an Edinburgh postnatal depression score of greater than or equal to 13 during their first year after childbirth were considered to be depression, and code “1” was given to them; those who scored less than 13 were not considered to have depression symptoms and coded as “0” (29).

*Known medical illness*: Women diagnosed with chronic medical illnesses like diabetes, cardiac disease, renal disease, hypertension, liver disease, and tuberculosis that are confirmed by doctors at health institutions are considered to have known medical illness (30).

*Decision-making power:* This was measured by the ability of women to act independently and decide on household activities including their health, their children’s health, freedom of movement, and control over finances without asking permission from another person. Depending on the items designed to assess maternal decision-making power, “2” was given if she decides by herself, “1” will be given if she decides jointly with her husband, and “0” was given if it is decided by others. Based on items designed to assess household decision-making power, women who scored above the median were considered to have good decision-making power and coded “1,” and those who scored below the median were considered to have poor decision-making power and were coded “0” (31).

*Maternal social support:* Social support was assessed by three items on the OSLO-3 social support scale. Mothers whose score was 3–8 were considered poor on social support and coded “0”. A score of 9–11 signified moderate social support and assigned the code “1”, and 12–14 indicated strong social support and code “2” was given (32).

*Postpartum Intimate partner violence (PIPV)*: This was assessed by 13 items developed from an adapted tool to assess domestic violence against women in low-income country settings. It includes physical violence, sexual violence, and psychological violence. Any mother who is the victim of at least one type of PIPV was coded “1” and who was not the victim was coded “0” (33).

*Maternal loneliness*: The University of California Los Angeles (UCLA-3) 20-item Loneliness Scale (Version 3) was used to measure maternal loneliness during the first year after childbirth. The UCLA-3 20-item Loneliness Scale was used to gather the total score. Based on this tool, code “0” was given for those mothers who scored <28 (no loneliness) and “1” was given for those who scored ≥28 (had loneliness) (34).

*History of adverse pregnancy outcome:* Mothers who have a history of abortion, neonates with congenital anomalies that are incompatible with life, stillbirth, and/or neonatal death were considered to have adverse pregnancy outcomes for the purpose of this study.

*Average monthly income*: Based on this income, the mother was categorized as living above or below the poverty line based on the current classification system by the World Bank group. Earning US$1.90 or 97.85 Ethiopian Birr (ETB) or lower per day meant living below the poverty line. At the current exchange rate of US dollar to Ethiopian Birr, for 30 days, earning 2935.5 ETB or lower indicated that the mother was living below the poverty line (35).

### Data collection instruments and procedures

In face-to-face interviewers, structured questionnaires prepared by Kobo Tool Box (an innovative open-source platform for collecting, managing, and visualizing data) was administered to collect the data. The tool has a socio-demographic component, reproductive history, maternal and child healthcare services, and psycho-social factors related to the mother. Postpartum intimate partner violence was assessed by 13 items containing sexual violence, psychological violence, and sexual violence (33). Postnatal depression was assessed by a validated Edinburgh postnatal depression tool that was validated in an Ethiopian context (29).

Maternal social support was assessed by a validated OSLO-3 social support scale that contains three items (32), and loneliness was assessed by the University of California Los Angeles (UCLA-3) 20-item Loneliness Scale (Version 3) (34). The outcome variable was assessed by the SHIS tool, which has 20 items to assess self-harm (27). Husband’s involvement in maternity and child healthcare was assessed by a tool developed from related literature that has nine items (28). Similarly, decision-making autonomy was assessed by nine items developed from the related literature (31).

### Data quality assurance

A pretest was done on 10% of the study participants one month before data collection at Wahama Hagalo Woreda. The internal consistency of the tool was checked and had a Cronbach’s α test of 0.79. Initially, the tool was developed in the English language and translated to Afan Oromo for actual data collection, and the Afan Oromo version was retranslated back to English to cross-check the consistency of the tool.

Data was collected from three diploma holder women midwifery health professionals and supervised by three diploma holder women nurses. Training was given for two days on the objectives of the study for data collectors and the training also included participant safety for both data collectors and supervisors. Supervision was carried out by supervisors daily for the sake of clarity, accuracy, and consistency of the data.

### Data processing and analysis

The data collected by Kobo Tool Box was exported to SPSS version 25 software for cleaning, coding, and further analysis. Descriptive statistics were done, and the results were presented using diagrams and tables. Both bivariable and multivariable logistic regression analyses were done to identify factors associated with self-harm during the first year after childbirth. Variables with a *p*-value <0.25 in binary logistic regression analysis were transferred to multivariable logistic regression.

The crude odds ratio (COR) and the adjusted odds ratio (AOR) with a 95% CI were calculated to show association and strength of association, respectively. In multivariable logistic regression analysis, variables with a *p*-value of <0.05 were reported as statistically significant. The model goodness-of-fit test was done. Finally, the results were presented both in the narrative, diagram, and table forms.

## Results

### Socio-demographic characteristics

Among the 423 postpartum mothers selected for this study, 415 mothers participated in the study, at a response rate of 98.10%. The study identified that 31.80% of the participants were in the 18–23 age category and 17.00% were in the 41–45 age category. As revealed by this study, 53.30% of the mothers were housewives and 57.38% of their husbands were farmers. In addition, 13.50% of mothers have no formal education, and 9.60% of their husbands have no formal education. Furthermore, 51.30% of mothers have an average monthly income below the poverty line, and 47.50% have family sizes greater than or equal to six (Table 1).

**Table 1:** Socio-demographic characteristics of the study participants.

Variables	Categories	Frequency	Percentages/100%
Age	18–23	132	31.80
	24–29	56	13.30
	30–35	66	15.90
	36–40	91	22.00
	41–45	70	17.00
Religion	Orthodox	151	36.40
	Protestant	146	35.20
	Muslim	102	24.50
	Others*	16	3.90
Maternal occupation	Housewife	221	53.30
	Merchant	130	31.30
	Government employed	64	15.40
Occupation of husband	Farmer	238	57.34
	Merchant	111	26.75
	Government employed	66	15.91
Maternal educational status	No formal education	56	13.50
	Primary education	151	36.40
	Secondary education	139	33.50
	Diploma and above	69	16.60
Husband educational status	No formal education	40	9.60
	Primary education	140	33.70
	Secondary education	148	35.70
	Diploma and above	87	21.00
Family size	Less than or equal to 3	95	22.90
	Four to five	123	29.60
	Greater than or equal to 6	197	47.50
Average Monthly income	Below poverty line	202	51.30
	Above poverty line	213	48.70

*Waqefata and Catholics.

### Medical, reproductive history, and substance-related factors

As identified by this study, 55.18% of the infants were girls, and 52.00% of the husbands were not satisfied with the gender of their infants. Similarly, 48.00% of mothers did not intend to have the baby during the index of pregnancy. Furthermore, 78.31 and 51.80% of the mothers have antenatal care (ANC) and postnatal care (PNC) follow-up, respectively. From the total study participants, 13.50% of mothers gave birth at home and 10.80% of them have a history of adverse pregnancy outcomes. It was revealed that only 45.10% of husbands were involved in maternity and child healthcare.

In addition, 5.10 and 3.62% of mothers have been diagnosed with medical illnesses and mental illnesses, respectively. Similarly, 2.66% of their husbands have been diagnosed with medical illnesses. Furthermore, 3.90% of mothers have a family history of known diagnosed mental illnesses. Out of the total study participants, 36.39% of mothers and 52.80% of their husbands were alcohol users. Finally, 15.90% of the husband participants consume the khat (Table 2).

**Table 2:** Medical, reproductive history, and substance-related factors of the study participants.

Variables	Categories	Frequency	Percentage/100%
Sex of infant	Female	229	55.18
	Male	186	44.82
Husband satisfaction to gender of the infant	Yes	195	48.00
	No	216	52.00
Maternal intention to have baby	No	201	48.40
	Yes	214	52.60
ANC follow-up	No	90	21.69
	Yes	325	78.31
PNC follow-up	No	200	48.20
	Yes	215	51.80
Place of delivery	Home	56	13.50
	Health institution	359	86.50
Husband involvement in maternity and child healthcare	No	228	54.90
	Yes	187	45.10
History of adverse pregnancy outcome	No	45	10.80
	Yes	370	89.20
Maternal known diagnosed medical illness	No	394	94.90
	Yes	21	5.10
Husband known diagnosed medical illnesses	No	404	97.34
	Yes	11	2.66
Maternal known diagnosed mental illnesses	No	400	96.38
	Yes	15	3.62
Maternal family history of known diagnosed mental problem	No	399	96.10
	Yes	16	3.90
Maternal alcohol use	Yes	151	36.39
	No	264	63.61
Husband alcohol use	Yes	219	52.80
	No	196	48.20
Husband’s khat use	Yes	66	15.90
	No	349	84.10

ANC, Antenatal care; PNC, Postnatal care.

### Psychosocial-related factors

Out of the total study participants, 13.50 and 32.30% of mothers suffered from postnatal depression and postpartum intimate partner violence. In addition, 41.90 and 17.10% of the mothers enjoyed poor and moderate social support, respectively, during their postnatal period. Similarly, 29.40% of the mothers were lonely and only 31.81% of the mothers have decision-making power over household activities (Table 3).

**Table 3:** Psychosocial-related factors.

Variables	Categories	Frequencies	Percentage/100%
Depression	No	359	86.50
	Yes	56	13.50
PIPV	No	281	67.70
	Yes	134	32.30
Social support	Poor	174	41.90
	Moderate	71	17.10
	Strong	170	41.00
Maternal decision-making power	Poor	283	68.19
	Good	132	31.81
Loneliness	No	293	70.60
	Yes	122	29.40

PIPV, postpartum intimate partner violence.

### Magnitude of self-harm among postnatal mothers attending infant immunization clinics

**Figure 2:**
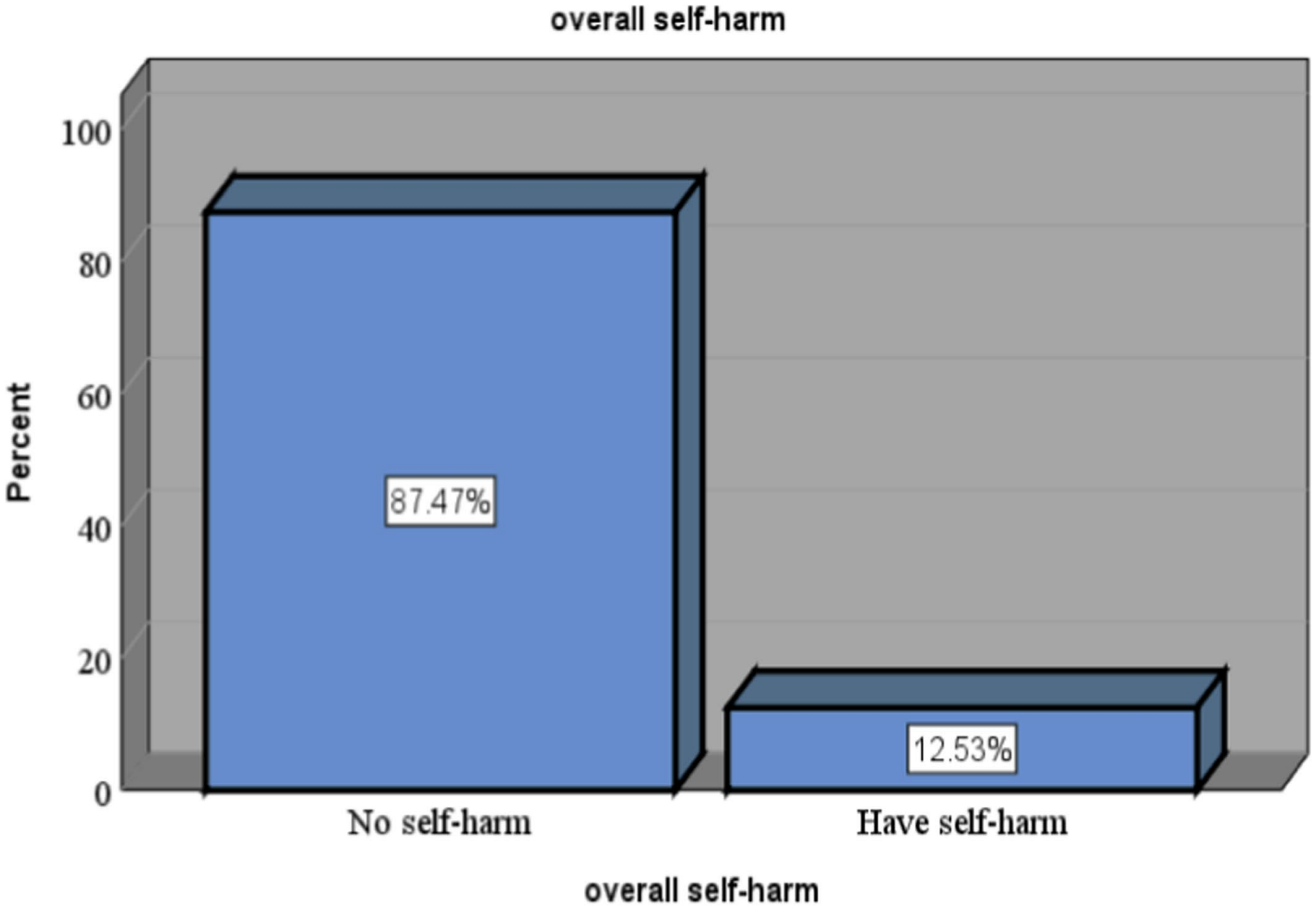
Magnitude of self-harm among mothers attending infant immunization clinics in Boneya Boshe Woreda.

This study revealed that the magnitude of self-harm among postnatal mothers in this study setting was 12.53% (95% CI: 9.33, 15.73) (Figure 2).

### Factors associated with self-harm and associated among postnatal mothers attending immunization clinics at public health institutions in Boneya Boshe Woreda, Western Ethiopia, 2023

From the total variables fitted for binary logistic regression at a *p*-value less than 0.25, six variables were significantly associated in the multivariable logistic regression model at a *p*-value less than 0.05 at a 95% CI. Variables significantly associated with self-harm in the final model were husband’s involvement in maternity and child healthcare, depression, loneliness, postnatal follow-up, postpartum intimate partner violence, and average monthly incomes.

This study identified that the odds of self-harm among mothers whose husbands do not participate in maternity and child healthcare were 1.90 times higher than those whose husbands participate in the care (AOR = 1.90; 95% CI: 1.12, 2.10). In addition, the odds of self-harm among mothers who have depression were 2.79 times higher than their counterparts (AOR = 2.79; 95% CI: 2.14, 6.94), and the odds of self-harm among mothers who do not have postnatal follow-up were 2.72 times higher than those who follow their postnatal care (AOR = 2.72; 95% CI: 1.28, 5.80).

Similarly, the odds of self-harm among mothers who have loneliness were 2.49 times higher than their counterparts (AOR = 2.49; 95% CI: 1.15, 5.40), and the odds of self-harm among mothers who suffered from postpartum intimate partner violence were 2.15 times higher than those who did not face the problem (AOR = 2.15; 95% CI: 1.01, 4.54). Finally, the study identified that the odds of self-harm among mothers with average monthly income below the poverty line were 3.70 times higher than those who were above the poverty line (AOR = 3.70; 95% CI: 2.17, 10.50) (Table 4).

**Table 4:** Factors associated with self-harm among postnatal mothers attending infant immunization clinics.

Variables	Categories	Magnitude of self-harm		COR(95%CI)	AOR(95%CI)
		Yes (52)	No (363)		
Husband involvement in maternity and child healthcare	No	36 (15.8%)	192 (84.2%)	2.00 (1.67,3.74)	1.9 (1.12,2.10)*
	Yes	16 (8.6%)	171 (91.4%)	1	1
Maternal intentions to have baby	No	41 (38.1%)	160 (44.1%)	4.73 (2.35,9.50)	3.76 (0.66, 4.51)
	Yes	11 (48.9%)	203 (55.9%)	1	1
Depression	No	34 (9.5%)	325 (90.5%)	1	1
	Yes	18 (32.1%)	38 (67.9%)	4.53 (2.33,8.80)	2.79 (2.14, 6.94)*
History of adverse pregnancy outcome	No	37 (10%)	333 (90%)	1	1
	Yes	15 (33.3%)	30 (66.7%)	4.50 (2.22,9.12)	2.69 (0.99,7.30)
Loneliness	No	24 (8.2%)	269 (91.8%)	1	1
	Yes	28 (23%)	94 (97%)	3.34 (1.84,6.05)	2.49 (1.15, 5.40)*
Postnatal follow-up	No	35 (17.5%)	165 (82.5%)	2.47 (1.34,4.57)	2.72 (1.28, 5.80)*
	Yes	17 (7.9%)	198 (92.1%)	1	1
Postpartum IPV	No	22 (7.8%)	259 (92.2%)	1	1
	Yes	30 (22.4%)	104 (77.6%)	3.40 (1.90,6.20)	2.15 (1.01, 4.54*)
Average monthly income	Below poverty line	45 (22.3%)	157 (77.7%)	4.41 (3.70, 9.20)	3.7 (2.17, 10.5)*
	Above poverty line	13 (6.13%)	200 (93.88%)	1	1
Social support	Poor	40 (23%)	134 (77%)	4.78 (2.30,9,91)	3.70 (0.59, 8.62)
	Moderate	11 (15.4%)	60 (84.5%)	2.93 (0.09,2.17)	0.34 (0.06, 1.87)
	Strong	10 (5.9%)	160 (94.1%)	1	1
Decision-making power	Poor	43 (15.3%)	238 (84.7%)	2.50 (1.18, 5.30)	2.30 (0.92,5.70)
	Good	9 (6.7%)	125 (93.3%)	1	1

*Significantly associated factors at *p*-value less than 0.05.

IPV, intimate partner violence.

## Discussion

Self-harm is a significant maternal health problem that can be prevented by screening and providing care for those mothers who have risks related to this problem. This study revealed that the magnitude of self-harm thought among postnatal mothers was 12.53%. The finding is comparable with the study conducted in Canada, which reported self-harm at 10.40% (19). However, the findings of this study were lower than those of the studies conducted in Sri Lanka (27.40%) (9), London (16.79%) (7), and the United States (19.3%) (36). The discrepancies might be due to differences in the assessment methods employed and sample size used for the studies, as these three studies used a larger sample size, which might increase the magnitude of self-harm thoughts.

Conversely, the findings of this study are lower than those of the studies conducted in northern Ethiopia (8.5%), South Africa (7%) (37), and Japan (9.1%) (38). The possible justifications for the discrepancies might be also attributed to the study period or years of the studies. The discrepancies between the study conducted in the northern part of Ethiopia and our study might be due to the current market inflation in Ethiopia, which might increase life expectancy and push mothers to self-harm as they are unable to fulfill the economic demands of their family members in the current market situation.

Husband’s involvement in maternity and child healthcare is significantly associated with self-harm thoughts during the postnatal period. The possible scientific reason might be that women whose husbands are involved in maternity and child healthcare feel confident that their husbands are with them in any ups and downs of life, which may affect their feelings as a result of physiological changes during childbirth (28). In addition, husbands who are involved in maternity and child healthcare consult the health professionals as they observe emotional and psychological changes in their wives during the postnatal period and have obtained information from health professionals regarding their families’ health (39).

Similarly, this study identified that having depression is significantly associated with self-harm thoughts. This evidence is supported by the study conducted in Canada (19). The possible reason might be that depression during the postpartum period pushes mothers to self-harm as a result of the unmanaged postpartum depression, which leads to postpartum psychosis, posing a high level of danger for both maternal and infant’s health, ending up with postpartum morbidity (7).

The study also revealed that loneliness is significantly associated with self-harm thoughts. Even though there was no evidence to support this finding, loneliness pushes the mothers to self-harm, and as a result, they may feel empty as they lack people around them during the postpartum period. If there are no people around them for support, the anxiety levels of mothers increase, which worsens the thoughts of self-harm as a defense mechanism to be out of their feelings, and loneliness leads to mental health problems (40).

In addition, the average monthly income is a significantly associated factor with thoughts of self-harm during the postnatal period. This finding is supported by the study conducted in northern Ethiopia (21). The reason might be that mothers whose monthly income is below the poverty line might be stressed about their income, which might not be sufficient to support the livelihood of the families. Similarly, they lose hope, which pushes them to take action on their lives if they are unable to fulfill their family’s needs due to lack of money to fulfill even their basic needs.

Furthermore, postpartum intimate partner violence is a significant factor associated with self-harm thoughts during the postnatal period. This finding is supported by the study conducted in the northern part of Ethiopia (21). The possible justification might be that mothers who suffer from this problem might fall into psychological crisis as a result of their husband’s bad attitude toward them, and those mothers lose hope, which pushes them to life-threatening problems like self-harm thoughts.

Finally, the study identified that postpartum follow-up is a significantly associated factor with maternal self-harm thoughts. As already known, postpartum follow-up is key for the upkeep of maternal and infant health and may help health professionals screen the problem early to take action. Postpartum follow-up is part of the maternal continuum of care, which improves overall maternal health (41). During postpartum follow-up, mothers may consult their health professionals for any mental health problems that may occur during the postpartum period as a result of hormonal and physiological changes related to childbirth.

### Limitations and strengths of the study

Since it was a cross-sectional study, it does not identify cause-and-effect relationships. In addition, since it was an institution-based study, it might lead to an underestimation of the magnitude of self-harm thoughts. The study focused on the neglected areas that hold great significance for reducing preventable causes of maternal deaths and assessed important factors that have a great impact on maternal health.

## Conclusion and recommendations

This study revealed that the magnitude of self-harm thoughts during the postnatal period was a significant maternal health problem in the study setting. It was identified that the husband’s involvement in maternity and child healthcare, loneliness, depression, average monthly income below the poverty line, postpartum follow-up, and postpartum intimate partner violence were factors significantly associated with maternal self-harm thoughts. Husband’s involvement in maternity and child healthcare is needed to overcome the problem. In addition, the Ministry of Health should develop an effective strategy for preserving maternal health after childbirth based on the identified factors. The future studies in this direction need to have better study designs and large sample sizes.

## Data availability statement

The raw data supporting the conclusions of this article will be made available by the authors, without undue reservation.

## Ethics statement

The ethical clearance was obtained from the Wallaga University Institute of Health Sciences. Written informed consent was obtained from the study participants after they were informed of the overall nature of the study and its benefits for the mothers and the implications for policymakers.

## Author contributions

LW: Conceptualization, Data curation, Formal analysis, Investigation, Methodology, Project administration, Resources, Software, Supervision, Validation, Visualization, Writing – original draft, Writing – review & editing, Funding acquisition. MH: Conceptualization, Investigation, Resources, Supervision, Writing – review & editing. ZA: Conceptualization, Investigation, Resources, Supervision, Writing – review & editing. GT: Conceptualization, Investigation, Resources, Supervision, Writing – review & editing. YW: Conceptualization, Investigation, Resources, Supervision, Writing – review & editing. WG: Conceptualization, Investigation, Resources, Supervision, Writing – review & editing. MAH: Conceptualization, Investigation, Resources, Supervision, Writing – review & editing. AG: Conceptualization, Investigation, Resources, Supervision, Writing – review & editing. ST: Conceptualization, Investigation, Resources, Supervision, Writing – review & editing. SA: Conceptualization, Data curation, Investigation, Methodology, Resources, Supervision, Validation, Visualization, Writing – review & editing.
